# Supplementary material for: A UMLS-based spell checker for natural language processing in vaccine safety
Source: BMC Med Inform Decis Mak. 2007 Feb 12;7:3. doi: 10.1186/1472-6947-7-3 (PMC1805499; doi:10.1186/1472-6947-7-3)
Supplement: Additional file 13 — RAP application directory. Sets up directory for RAP (RDF application for PHP) source code [file 1472-6947-7-3-S13.gz › rap/doc/phpdoc/__filesource/fsource_resModel__resModelResLiteral.php.html]

xml version="1.0" encoding="iso-8859-1"?


File Source for ResLiteral.php


# Source for file ResLiteral.php

Documentation is available at ResLiteral.php

```
1. <?PHP
2. // ----------------------------------------------------------------------------------
3. // Class: ResLiteral
4. // ----------------------------------------------------------------------------------


8. /**
9. * An RDF literal.
10. * The literal supports the xml:lang and rdf:datatype property.
11. * For XML datatypes see: http://www.w3.org/TR/xmlschema-2/
12. *
13. *
14. * <BR><BR>History:<UL>
15. * <LI>10-01-2004                : First version of this class.</LI>
16. *
17. * @version  V0.9.1
18. * @author Daniel Westphal <mail at d-westphal dot de>
19. *
20. * @package resModel
21. * @access    public
22. ***/

24. class ResLiteral extends Literal
25. {
26. /**
27. * Holds a reference to the associated model
28. * @var        ResModel
29. * @access    private
30. */
31. var $model;


34. /**
35. * Constructor
36. * You have to supply a string.
37. *
38. * @param    string    $str        label of the literal
39. * @param     string $language    optional language identifier
40. */
41. function ResLiteral($str,$language = null)
42. {
43. parent::Literal($str,$language);

45. }

47. /**
48. * Sets the reference to the assocoated model.
49. *
50. * @param    object model    $model
51. * @access public
52. */
53. function setAssociatedModel(& $model)
54. {
55. $this->model=& $model;
56. }

58. /**
59. * Get the reference to the assocoated model.
60. *
61. * @return    object model    $model
62. * @access public
63. */
64. function & getAssociatedModel()
65. {
66. return $this->model;
67. }

69. }
70. ?>
```

Documentation generated on Thu, 7 Jul 2005 13:43:38 +0200 by phpDocumentor 1.3.0RC3
